# Genomic, Habitat, and Leaf Shape Analyses Reveal a Possible Cryptic Species and Vulnerability to Climate Change in a Threatened Daisy

**DOI:** 10.3390/life11060553

**Published:** 2021-06-11

**Authors:** Colette Blyth, Matthew J. Christmas, Douglas C. Bickerton, Martin F. Breed, Nicole R. Foster, Greg R. Guerin, Alex R. G. Mason, Andrew J. Lowe

**Affiliations:** 1 School of Biological Sciences, University of Adelaide, Adelaide 5005, Australia; nicole.foster@adelaide.edu.au (N.R.F.); greg.guerin@adelaide.edu.au (G.R.G.); a1741421@student.adelaide.edu.au (A.R.G.M.); andrew.lowe@adelaide.edu.au (A.J.L.); 2 Department of Medical Biochemistry and Microbiology, Uppsala University, 751 23 Uppsala, Sweden; 3 Independent Researcher, Adelaide 5052, Australia; dugalugs29@gmail.com; 4 College of Science and Engineering, Flinders University, Bedford Park 5042, Australia; martin.breed@flinders.edu.au; 5 Terrestrial Ecosystem Research Network (TERN), University of Adelaide, Adelaide 5005, Australia

**Keywords:** *Olearia pannosa*, conservation genomics, habitat suitability, genetic diversity, gene flow, cryptic species

## Abstract

*Olearia pannosa* is a plant species listed as vulnerable in Australia. Two subspecies are currently recognised (*O. pannosa* subsp. *pannosa* (silver daisy) and *O. pannosa* subsp. *cardiophylla* (velvet daisy)), which have overlapping ranges but distinct leaf shape. Remnant populations face threats from habitat fragmentation and climate change. We analysed range-wide genomic data and leaf shape variation to assess population diversity and divergence and to inform conservation management strategies. We detected three distinct genetic groupings and a likely cryptic species. Samples identified as *O. pannosa* subsp. *cardiophylla* from the Flinders Ranges in South Australia were genetically distinct from all other samples and likely form a separate, range-restricted species. Remaining samples formed two genetic clusters, which aligned with leaf shape differences but not fully with current subspecies classifications. Levels of genetic diversity and inbreeding differed between the three genetic groups, suggesting each requires a separate management strategy. Additionally, we tested for associations between genetic and environmental variation and carried out habitat suitability modelling for *O. pannosa* subsp. *pannosa* populations. We found mean annual maximum temperature explained a significant proportion of genomic variance. Habitat suitability modelling identified mean summer maximum temperature, precipitation seasonality and mean annual rainfall as constraints on the distribution of *O. pannosa* subsp. *pannosa*, highlighting increasing aridity as a threat for populations located near suitability thresholds. Our results suggest maximum temperature is an important agent of selection on *O. pannosa* subsp. *pannosa* and should be considered in conservation strategies. We recommend taxonomic revision of *O. pannosa* and provide conservation management recommendations.

## 1. Introduction

Populations of threatened plant species are typically small and fragmented, suffering low genetic diversity associated with increased genetic drift and elevated inbreeding rates [1,2,3,4,5]. Additional to these factors, a changing climate places increasing pressure on threatened populations. Plants can respond in one of three ways in the face of climate change: adapt to the new environment, migrate to locations with suitable conditions, or perish [6,7]. In this era of accelerated climate change, plants may not have the capacity to respond through in-situ adaptation, particularly those already suffering loss of genetic diversity [8,9,10,11,12]. Furthermore, the sheer rate of climate change combined with limitations to migration and dispersal means that for many plant populations the likelihood of migrating at a suitable pace is low [13]. 

For plants, natural dispersal generally occurs via distribution of seed and pollen. For threatened plant species, however, undirected natural dispersal is likely insufficient to ensure persistence of, and gene flow among, populations [14]. In these scenarios, conservation interventions, such as facilitated gene flow, are now being considered to minimise extinction risks [15,16]. Various approaches to seed sourcing can be used, such as facilitating gene flow between fragmented populations to maximise overall genetic diversity, increasing gene flow to designated populations (e.g., to encourage adaptation to predicted climate change scenarios), and sourcing seed entirely from non-local provenances where the populations are already presumed to be well adapted to predicted future climate [1,16,17,18,19,20,21,22,23]. However even with such interventions, environmental change may take place more quickly than a species’ capacity to adapt. In extreme scenarios, habitat suitability is expected to change considerably under climate change, with predications for some species showing minimal overlap between current and future suitable habitat [24,25]. In such instances, when extinction risk is high and unlikely to be alleviated by facilitated gene flow alone, establishing new populations through assisted migration (moving a species to new, more suitable habitat) and range expansion strategies can provide a last resort opportunity for conservation management [26,27,28]. 

The genus *Olearia* (Moench) is widespread across Australasia and New Guinea and there are roughly 130 species of Olearia in Australia, making it the country’s largest Asteraceae genus [29]. However, many species within this genus require further investigation into their taxonomy and phylogeny. This is highlighted by the number of recent studies describing or reinstating Olearia species from both molecular and taxonomic data [29,30,31,32,33,34]. *Olearia pannosa* (Hook) is a long-lived understory daisy shrub, endemic to southeastern Australia. It generally occurs in small and fragmented stands in areas that have been converted to agriculture over the last 150 years and is classed as nationally vulnerable [34]. Significant investments have been made to conserve the species, and one of the priority conservation actions is to establish new translocated populations resilient to climate change [35]. 

**Figure 1:**
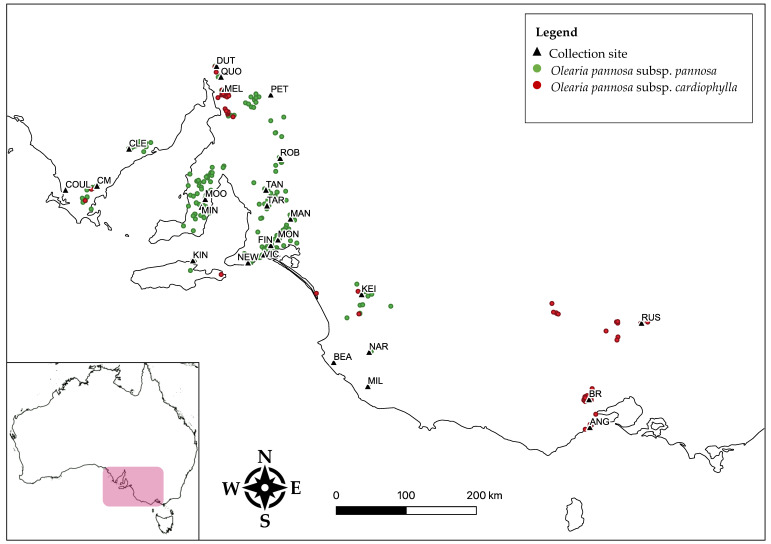
Study collection sites (black triangles) and occurrence records of *O. pannosa* subsp. *pannosa* (green markers) and *O. pannosa* subsp. *cardiophylla* (red makers) (Atlas of Living Australia occurrence downloads at https://biocache.ala.org.au/occurrences/search?q=qid:1609477224810 and https://biocache.ala.org.au/occurrences/search?q=qid:1609477191724, both accessed on 1 January 2021).

*Olearia pannosa* has undergone several taxonomic revisions [34]. Currently, two subspecies are described based on leaf shape differences: *O. pannosa* subsp. *pannosa* (Cook), which is widespread across southern South Australia and has small, narrow leaves; and *O. pannosa* subsp. *cardiophylla* (F. Muell) which has larger, more heart-shaped leaves and is only found in isolated stands in the Flinders Ranges of South Australia (where it overlaps with *O. pannosa* subsp. *pannosa*) and in several isolated populations in Victoria (Figure 1). Historically, *O. pannosa* subsp. *cardiophylla* was considered a separate species and was even once within a different genus [34]. Currently there is no information on the genetic relatedness between *O. pannosa* subsp. *pannosa* and subsp. *cardiophylla*, nor on levels of genetic divergence between the Flinders Ranges *O. pannosa* subsp. *cardiophylla* populations and those in the southeast of South Australian and Victoria. Furthermore, there is a lack of understanding of the relationship between genetic divergence and leaf shape variation, which could assist practitioners with management outcomes. Therefore, to delineate a suitable conservation strategy for *O. pannosa*, we first need to understand the genetic and leaf shape differences between the various populations and subspecies.

Here, we adopt a multidisciplinary approach that combines genome-wide population genetic marker data, leaf shape and habitat suitability modelling to obtain a high-resolution picture of population structure, divergence, and diversity within this species. We aimed to test for genetic and leaf shape differences among the recognised subspecies and clarify subspecies boundaries. In particular, we aimed to reveal the relationship between isolated *O. pannosa* subsp. *cardiophylla* collection localities in the Flinders Ranges of semi-arid South Australia and those located further east. For *O. pannosa* subsp. *pannosa*. We also sought to assess whether any of the genetic and phenotypic diversity among the collection localities associates with environment and used habitat suitability modelling to reveal environmental limitations to the species’ distribution. We discuss the outcomes from these analyses in terms of their implications for the conservation management.

## 2. Materials and Methods

### 2.1. Study Species and Sampling

*Olearia pannosa* is a low shrub (~1.5 m) which spreads by producing root suckers [34]. The species can be found in a range of floristic communities and climatic conditions within southeastern Australia. There are currently two subspecies with similar ecology but can be differentiated by leaf shape: *O. pannosa* subsp. *pannosa* (silver daisy bush), and *O. pannosa* subsp. *cardiophylla* (velvet daisy bush). *O. pannosa* subsp. *pannosa* is listed as vulnerable under the Australian Government’s Environment Protection and Biodiversity Conservation Act 1999 [36]; and vulnerable under the South Australian National Parks and Wildlife Act 1972 [37]. *O. pannosa* subsp. cardiophylla is also of conservation concern and has been listed as vulnerable under the Victorian Advisory List of Rare or Threatened Plants in Victoria—2014 [38].

### 2.2. Study Species and Sampling

**Figure A1:**
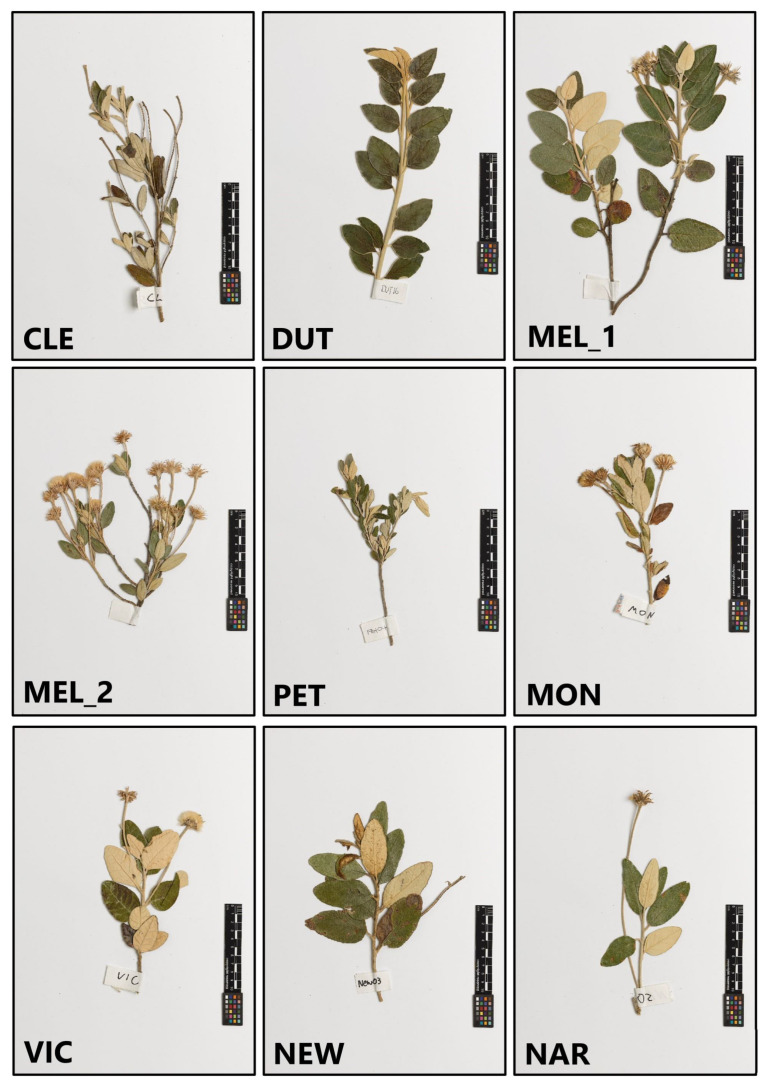
Examples of vouchers used in the leaf shape analysis. Vouchers selected represent the range of variation found across collection localities assigned to each genetic group. *Olearia* (FR) examples are from Melrose (MEL_1) and Dutchmans Stern (DUT). *O. pannosa* subsp. *pannosa* is represented by the collection localities Cleeves (CLE), Melrose (MEL_2), and Peterborough (PET). Victor Harbor (VIC) and Newland Head (NEW) are both localities which have admixture between *O. pannosa* subsp. *pannosa* and *O. pannosa* subsp. *cardiophylla.* The *O. pannosa* subsp. *cardiophylla* example is from Naracoorte (NAR).

Leaf samples were collected from a total of 191 individuals from 25 localities spanning the ranges of both subspecies (Figure 1, Table A1). Each sample (individual plant) consisted of five leaves: one leaf collected from each aspect/side of the plant (North, South, East, West), and one from the central apex point of the plant—generally the highest/near highest growing point of the plant. Nearest neighbour individuals were avoided to minimise relatedness between genotyped individuals and obvious clones and root suckers were avoided. Samples were dried in a sealed container with silica beads. Collections were made during winter and spring of 2018. Voucher specimens were also collected at each locality (except the Victorian stands), and where there was evidence of morphological variation between individuals at a site, multiple specimens were collected. Voucher specimens consisted of a 20–30 cm small branch taken from a growing point of the plant, which was labelled and pressed at the earliest convenience (Figure A1). Vouchers were submitted to the State Herbarium of South Australia, and an identification was provided for each collection locality (Table A1).

### 2.3. DNA Extraction, Sequencing, and Filtering

All DNA extraction, library preparation and sequencing were conducted at the Australian Genome Research Facility (Adelaide, Australia). The Machery-Nagel Nucleospin Plant II Kit was used to extract DNA prior to undergoing double digest restriction-associated DNA sequencing (ddRADseq) [39]. The restriction enzymes Pstl and MseI were used for the digestion, then barcoded adaptors were ligated to the restriction site overhangs for each sample. Samples were then pooled, and the DNA size selected using Blue Pippin (Sage Science, Beverly, MA, USA) (Wide) to retain fragments 280–342 bp. The resulting library was amplified via Polymerase Chain Reaction (PCR) for 11 cycles using indexed primers. Libraries were assessed by gel electrophoresis (D1000 ScreenTape Assay, Agilent, CA, USA), quantified by qPCR (KAPA Library Quantification Kits for Illumina, Roche, Basel, Switzerland), and then single read sequenced (150 bp) on the NextSeq 500 system using NextSeq 500 high Output Kit v2 reagents (150 cycles).

A total of 637,395,304 sequences were generated across 192 *O. pannosa* samples and we processed these using the STACKS pipeline [40,41] at AGRF (Melbourne, Australia). Reads were de-multiplexed using the unique sample adapter barcodes and filtered for both read quality and the presence of a restriction site. The resulting FASTQ files were then trimmed to the size of the shortest read, minus 2 bp to account for differences in read length due to any variation in barcode length. Reads were then stacked according to similarity, forming tags of reads. Tags which appear across all samples were collated (catalogue tags), and genotypes were then allocated to the common polymorphic sites. The collated SNPs across all individuals were then filtered using the following settings: minimum number of reads required at a stack to call a homozygous genotype = 5; minor allele frequency, below which a stack is called a homozygote = 0.05; minimum minor allele frequency to call a heterozygote = 0.1 (above 0.05 but below 0.1, a stack is called ‘unknown’). Computed genotypes were then output as a single VCF file (Supplementary File S1) for further filtering and analysis.

We used VCFtools [42] to filter SNPs with a minor allele frequency of less than 0.1 and with a depth of coverage of less than10 reads per individual. We removed SNPs with >25% missing data across all individuals and individuals with >25% missing SNP calls. We then used PLINK [43] to identify SNPs in linkage disequilibrium with the ‘indep-pairwise’ command, with one SNP in a pair of linked SNPs (r^2^ > 0.8) being removed from the dataset. 

Levels of ploidy were assessed by examining the distribution of allele dosage at heterozygous sites. For this, the counts of reads supporting each allele at a heterozygous site were extracted from the VCF file using a custom Perl script, and each individual’s distribution was plotted. True allelic proportions for diploids, triploids and tetraploids at heterozygous sites are 1:1 (0.5), 1:2 or 2:1 (0.33 or 0.66), and 1:3, 2:2, or 3:1 (0.25, 0.5, or 0.75), respectively. No sample deviated from a normal distribution centred on a frequency of 0.5, suggesting that all samples are diploid.

For each SNP across all samples, inbreeding coefficient (*F*) values were estimated using the G-stats calculator in GENODIVE v. 3.2 [44,45]. As negative *F* indicates greater than expected heterozygosity under Hardy-Weinberg equilibrium, and could be indicative of paralogous reads being stacked together in the STACKS pipeline [46], we used VCFtools to remove SNPs with significantly negative *F* (significance assessed using permutation tests with 10,000 permutations and a significance threshold of *p* < 0.05).

After the filtering steps, 175 individuals and 9997 SNPs remained. We used this dataset to perform the PCA, DAPC, and ADMIXTURE. After filtering for highly related individuals, 169 individuals and 9997 SNPs remained in the dataset. We used this dataset to perform *F_ST_*, genetic diversity and inbreeding statistics, and AMOVA analyses.

### 2.4. Genetic Structure Analysis

We then analysed the population genetic structure across all samples in the filtered SNP. To identify the most likely number of genetic clusters (K), we used two different genetic clustering analyses. First, we used the non-model-based Discriminant Analysis of Principal Components (DAPC) of the R package adegenet [47]: genetic data were transformed into uncorrelated components using principal component analysis (PCA). The number of genetic clusters was then defined using k-means, a clustering algorithm that looks for the value of K that maximises the variation between groups. The Bayesian Information Criterion (BIC) was calculated for K = 1–20, and the K value with the lowest BIC suggesting the optimal number of clusters. A discriminant analysis was then performed on the first 80 principal components with the function ‘dapc’, implemented in R, in order to efficiently describe the genetic clusters and assign samples to each cluster. We also used ADMIXTURE [48], a model based genetic clustering algorithm, to estimate the most likely number of clusters (K) in our dataset. ADMIXTURE was also run for K values 1–10 and the most likely value of K was assessed by comparing the cross-validation errors (cv errors) between runs, with the lowest cv error indicating greatest support. We repeated each ADMIXTURE analysis ten times and used CLUMPAK to combine the results and construct bar plots of individual assignment to clusters for the most supported values of K using DISTRUCT [49,50]. We then calculated pairwise *F_ST_* between all collection localities using the R package StamPP [51]. This was then exported to Splitstree to create a neighbour-joining (NJ) tree [52]. 

### 2.5. Kinship, Genetic Diversity, and Inbreeding Analysis

The dataset was filtered for all first-degree relations (parent-siblings and sibling-siblings) following methods outlined in Dutheil [53] to avoid these influencing our statistical results. The king-cutoff option in PLINK 2 [54] was used to remove one in a pair of samples with a kinship coefficient greater than or equal to 0.177 (6 individuals were removed, Table A2 and Table A4). A kinship coefficient of 0.25 corresponds to first-degree relations, so the 0.177 threshold should capture all of these. This step should also have removed any likely clones from the dataset, which was an important consideration given *O. pannosa* is known to reproduce clonally. We then used PLINK 2 [54] to calculate pairwise kinship between individuals. We then calculated average kinship within each locality in R v.4.0.4 As the genetic structure analysis identified such strong genetic differentiation between three of the clusters, we separated the dataset according to these groupings. Each dataset was refiltered for minor allele counts of 1 with VCFtools [39] to remove non-variants for subsequent analyses (See Table A2 for number of variants remaining at each collection locality). 

For the collection localities in each of the three genetic groups, we separately estimated the inbreeding coefficient (*F*), and observed (H_O_) and expected (H_E_) heterozygosity in GENODIVE v3.2 [44,45]. For the collection locality COUL (COULTA), there were insufficient samples to calculate these statistics and so this collection locality was not included in this analysis. A one-way ANOVA and Tukey pairwise comparisons were used to assess group differences in kinship, F, H_O_ and H_E_ using R v.4.0.4 [55]. To assess the distribution of genetic diversity amongst and within the genetic groups, we conducted a nested Analysis of Molecular Variance (AMOVA) using GENODIVE v3.2 to explore genetic variation within individuals, among collection localities nested within groups, and among groups. Further AMOVA analyses were then run separately for *O. pannosa* subsp. *pannosa* and *O. pannosa* subsp. *cardiophylla* (there was not enough data to complete the analysis for *Olearia* (FR)) to compare how genetic variation was distributed within individuals and among collection localities between these two genetic groups.

### 2.6. Leaf Trait Analysis

In order to investigate the environmental influence on leaf shape and the variation that exists between collection localities, leaf traits were identified and analysed. In total 110 individuals were measured from across 22 collection localities (note that Victorian collection localities were excluded from this component of the study as we did not have suitable material for the measurements). Leaf traits were based on the methods of Lowe and Abbott [56] and measured using ImageJ software v 1.53e (https://imagej.nih.gov/ij/download.html, accessed on 10 November 2020) [57] from photo images of voucher samples (See Figure A1 for examples). Three measurements were taken; leaf length (L) (measured from leaf base to apex), leaf width (W) (measured at the widest point of leaf), and the distance along the midrib to the widest point of the leaf from the base of the leaf, giving width location (WL). Leaf area measurements were square root transformed to satisfy the requirement of normality of residuals. Leaf length (L), width (W) and the distance at which the width measurement was taken from the base (WL) were used to estimate leaf area (LA), employing the formula

LA = (L × W / 2) + ((L − WL) × 2 / 2)(1)

We estimated leaf ovality or roundness by dividing the length along leaf mid rib to widest point of the leaf by total leaf length. A value around 0.5 indicates an oval leaf, <0.5 indicates a more heart-shaped leaf, and >0.5 a ‘gingko’ shaped leaf. The ratio of leaf length to leaf width (calculated as leaf length divided by width), was also determined. Where possible, five leaves were measured from each voucher specimen, with five individuals sampled from collection locality. A standardised leaf sampling protocol was conducted by sampling the first mature leaf from the base of the specimen, with subsequent leaves sampled working towards the apex. In some circumstances direct leaf measurement was not possible due to folding or damage, in which case width was calculated by measuring distance from leaf edge (at widest point) to leaf midrib and multiplying by two.

A principal component analysis (PCA) was performed using the ‘prcomp’ function in R v.4.0.4 [55] on the measurements for all measured leaf samples to assess differences in leaf shape among the three genetic clusters. Trait values were centred and scaled. One-way ANOVA and Tukey pairwise comparisons were used to assess group differences in principal component axes 1 (PC1) and 2 (PC2) using R v.4.0.4 [55].

### 2.7. Redundancy Analyses—Both Genetic Data and Leaf Variation

To assess the degree to which the environment explains observed genetic and phenotypic variation, partial redundancy analyses (RDA) were performed for *O. pannosa* subsp. *pannosa* samples. We focused on this genetic group as they were distributed over a wide environmental gradient and by omitting *O. pannosa* subsp *cardiophylla* samples confounding genetic structure can be removed from the analysis. A set of 44 environmental (e.g., climate, soil, and landscape) variables were sourced from stacks of 9-s resolution (i.e., approximately 250 m pixels) rasters [58,59,60]. Environmental values were extracted for each raster at the coordinates of collection localities of *O. pannosa* subsp. *pannosa* using the RASTER analytical package in R [61]. A subset of these extracted environmental variables with low collinearity were assessed using the ‘cor’ function in R v.4.0.4 [55]. They were: mean annual maximum temperature (°C), mean annual minimum temperature (°C), annual precipitation (mm), total nitrogen (%), total phosphorus (%), organic carbon (%), and elevation (m).

Redundancy analyses (RDA) were performed on (a) the allele frequencies of all SNPs in collection localities of *O. pannosa* subsp. *pannosa* and (b) leaf trait measurements for all samples of *O. pannosa* subsp. *pannosa* in order to assess how much of the genetic and phenotypic variation among our collection localities can be explained by spatial (i.e., resulting from isolation by distance) and environmental (i.e., indicating selection) factors (Supplementary File S2) using the VEGAN analytical package v2.5-6 in R [62]. First, spatial coordinates for (a) each sampling stand or (b) each sample were centred and converted into third degree polynomials. An RDA of allele frequencies-all spatial polynomials was then performed using the ‘rda’ function. The ‘ordistep’ function was then used to carry out forward stepwise model building for the RDA, adding in one polynomial to the model at a time and assessing significance using permutation tests. This revealed x, y, and x^2^ to be significant contributors to the model and as such only these variables were retained for the RDA. The variance between the selected spatial and all environmental variables was assessed using the ‘varpart’ function and significance of the partitioning was assessed using an analysis of variance (ANOVA)-like permutation test for RDA with the ‘anova.cca’ function. In each case, we tested whether any of the genetic or phenotypic variance was significantly explained by environmental variables by running an RDA on the models (a) ‘allele frequencies-environmental variables conditioned on spatial variables’ and (b) ‘leaf traits-environmental variables conditioned on spatial variables’. To assess which of the environmental variables provided the most explanatory power in the models, we performed the same stepwise model building described above for the spatial variables on the environmental variables.

### 2.8. Habitat Suitability Modelling

For the largest genetic group, *O. pannosa* subsp. *pannosa,* MaxEnt and Random Forest were used to assess which environmental variables best explain habitat suitability given the species’ current distribution. Occurrence data were sourced from collection locality sampling records, consisting of 105 locations. We modelled current distribution using the BCCVL online tool (http://www.bccvl.org.au/, accessed on 25 February 2021); Ref. [63] by statistically comparing confirmed presence sites against predictor spatial layers. Predictors included national soil data provided by the Australian Collaborative Land Evaluation Program ACLEP (www.clw.csiro.au/aclep, accessed on 25 February 2021), and a current Australian climate baseline of 1976 to 2005 provided as bioclim layers by BCCVL [64].

Distributions were predicted based on current climate and soil using MaxEnt and Random Forest, which are widely used machine-learning algorithms for mapping habitat suitability [65,66]. Models were run using 10,000 pseudo-absences confined to the land area within 250 km of a convex polygon surrounding presence locations. Starting with the full set of potential variables included in the edaphic and climatic layer sets, we sequentially removed both highly correlated (i.e., r > 0.8) and poorly performing predictors contributing to 1% or less of the model.

## 3. Results

### 3.1. Genetic Structure Analysis

**Figure 2:**
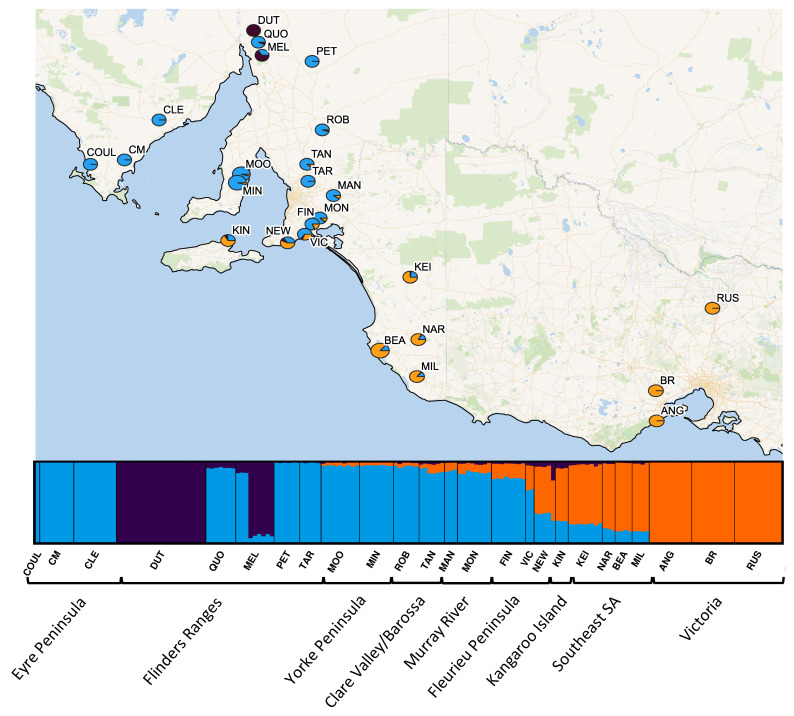
Individual genetic cluster assignment based on ADMIXTURE results. Pie charts at each sampling site show the overall proportion of individuals assigned to the two genetic clusters (blue generally aligns with collection localities identified as *O. pannosa* subsp. *pannosa* group, purple with *Olearia* (FR), and orange with *O. pannosa* subsp. *cardiophylla.* Individual assignments are represented in the bar plots at the bottom of the map.

**Figure 3:**
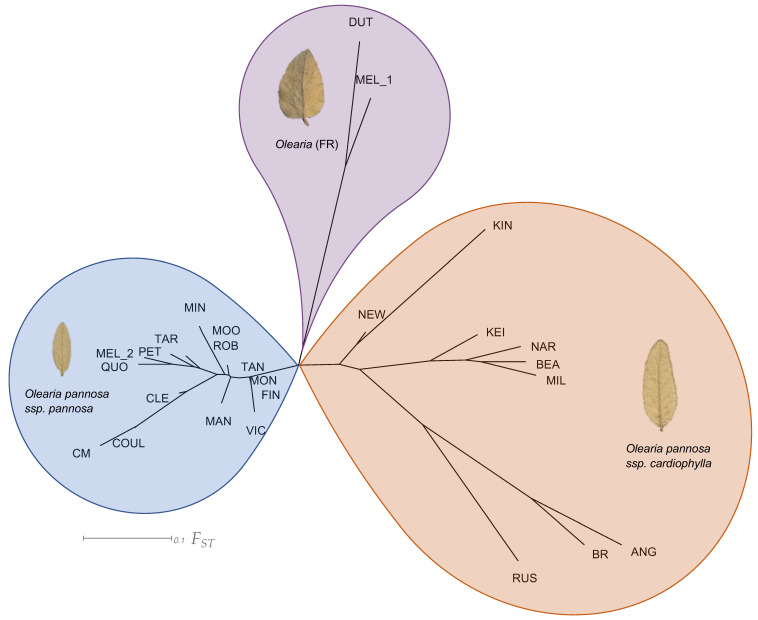
Neighbour joining tree of pairwise genetic differentiation *(F_ST_*) between collection localities. Colours represent genetic cluster assignment identified in the ADMIXTURE and DAPC analyses (blue: collection localities identified as *O. pannosa* subsp. *pannosa* group, purple: *Olearia* (FR), orange: *O. pannosa* subsp. *cardiophylla*). Images of leaves represent typical shape found in each genetic cluster. The *O. pannosa* subsp. *cardiophylla* cluster leaf is from Naracoorte (NAR), the *Olearia* (FR) cluster from Melrose (MEL), and the *O. pannosa* subsp. *pannosa* cluster leaf is also from Melrose (MEL). Note that as the site Melrose (MEL) contains individuals belonging to different genetic clusters, those belonging to *Olearia* (FR) have been labelled as MEL_1 and *O. pannosa.* subsp. *pannosa* have been labelled as MEL_2 (see Table A3 for full output and Figure A1 for images of full voucher specimens).

**Figure A2:**
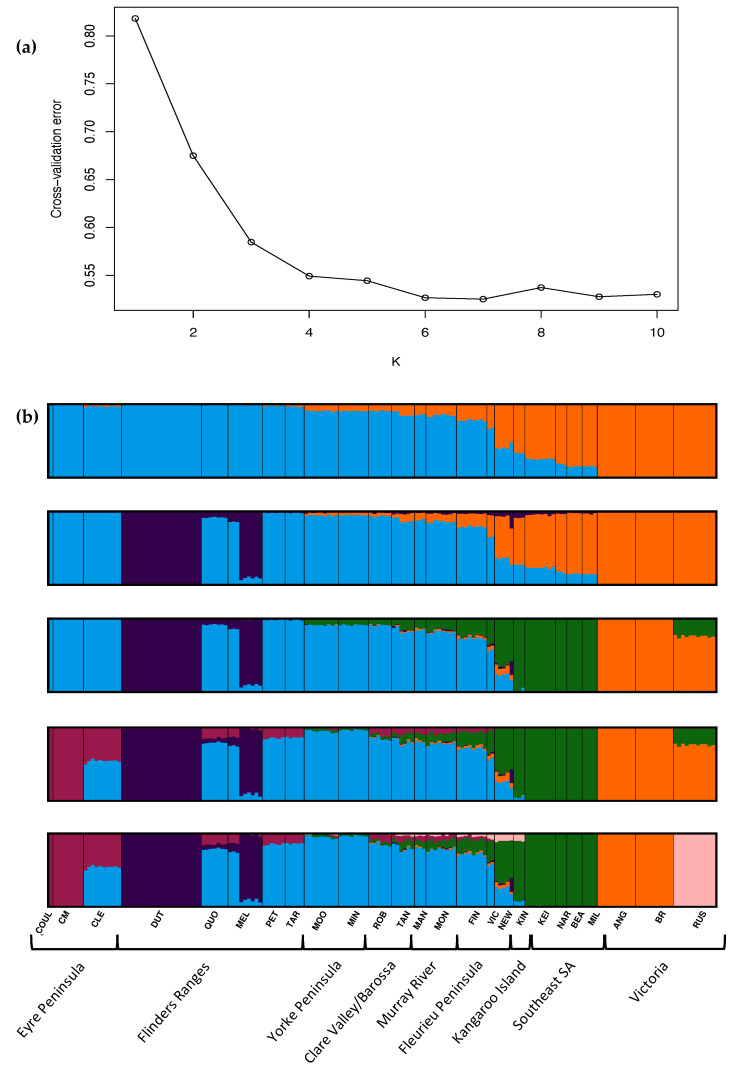
(**a**) ADMIXTURE error results—the cv errors are plotted for K = 1–10, with the lowest cv error indicating greatest support at K = 6. (**b**) Bar plots represent individual genetic cluster assignment from ADMIXTURE results from K = 2 to K = 6 (after which the assignment becomes messy and incomprehensible).

**Figure A3:**
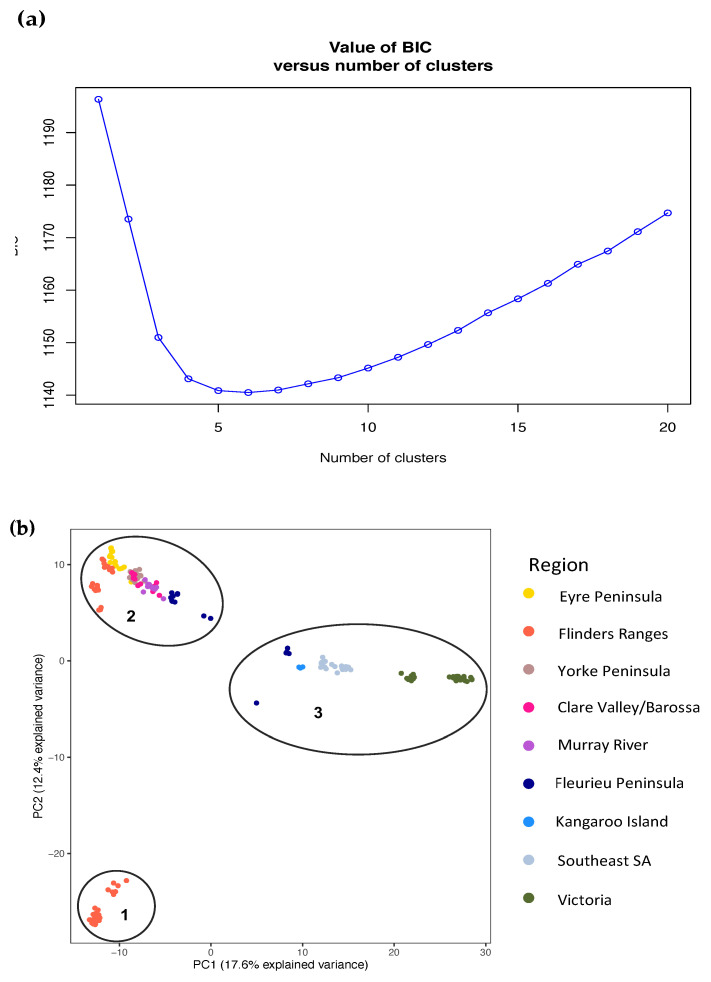
(**a**) Bayesian Information Criterion (BIC) values for each number of clusters, with the lowest BIC indicating support for five or six clusters; (**b**) Principal Component Analysis showing population genetic structure across all samples. Colours represent regions and ellipses identify the genetic clusters (1–3) identified in the DAPC analysis for K = 3.

Strong genetic structure was identified across the range of *O. pannosa* and was supported by all genetic analyses (Figure 2, Figure 3, Figure A2 and Figure A3, Table A3). While the BIC criterion from the DAPC analysis (Figure A3) and CV error from the ADMIXTURE (Figure A2) both indicate that K = 5–6 is the most likely value of K, there is strong evidence of three key genetic groups within *O. pannosa* which have genetic barriers that cannot be explained by geographic isolation (Figure 2). This was shown in samples from the Flinders Ranges in semi-arid South Australia identified as *O. pannosa* subsp. *cardiophylla*. These were genetically distinct from all other samples, even when collected from the same geographic location (see collection site Melrose (MEL) in Figure 2, Figure 3, Figure A1 and Figure A2, Table A3). The second such genetic break is found on the Fleurieu Peninsula of South Australia. Despite evidence of admixture in these collection localities (Figure 2), the geographically proximate Newland Head (NEW) and Victor Harbor (VIC) (~12 km apart) fall into different clusters in the PCA and different genetic groups in the DAPC analysis (Figure 2, Figure 3 and Figure A3, Table A1 and Table A3). 

We therefore present three key genetic groups, acknowledging that there is further substructure within them. These groups are:

1. The southern Flinders Ranges samples identified by the State Herbarium of South Australia as *O. pannosa* subsp. *cardiophylla* (Table A1, Peter Lang, pers. comm.). From here on, we will refer to this group as *Olearia* (FR).
2. Samples from Coulta (COUL) on the Eyre Peninsula to Victor Harbor (VIC) on the Fleurieu Peninsula (excluding the *Olearia* (*FR*) specimens collected at Dutchman’s Stern (DUT) and Melrose (MEL)) were mainly identified as *O. pannosa* subsp. *pannosa* (State Herbarium of South Australia, P. Lang 2021, personal communication; Table S1). There is evidence of some substructure within this group, with geographically isolated sites Coulta (COUL) and Cummins (CM) on the Eyre Peninsula forming a distinct cluster. Notably, there was some admixture between genetic groups, particularly in the samples collected at Victor Harbor (VIC), which were identified as *O. pannosa* subsp. *cardiophylla* (Table A1). From here on this genetic group will be referred to as *O. pannosa* subsp. *pannosa.*

All other samples ranging from the NEW (Newland Head) collection locality on the Fleurieu Peninsula across to the Victorian collection localities were identified as *O. pannosa* subsp. *cardiophylla* (State Herbarium of South Australia, P. Lang 2021, personal communication; Table A1). However, admixture was also detected in several collection localities geographically close to stands of *O. pannosa* subsp. *pannosa* (e.g., Newland Head (NEW)). From hereon we will call this genetic group *O. pannosa* subsp. *cardiophylla.*

Across the whole dataset, our analysis of pairwise genetic differentiation (*F_ST_*) showed a considerable range of differentiation between collection localities (*F_ST_*_=_ 0.00–0.75; Figure 3, Table A3). Within the *Olearia* (FR) group (DUT & MEL_1), there was a moderately high level of genetic differentiation (*F_ST_* = 0.23). Within *O. pannosa* subsp. *pannosa*, apart from collection localities on the Eyre Peninsula, genetic differentiation between collection localities was generally low (*F_ST_* = 0.00–0.13). On the Eyre Peninsula, however, collection localities displayed a greater range of differentiation (*F_ST_* = 0.05–0.30) reflecting their more isolated locations. There was considerable variation in the levels of genetic differentiation between collection localities in *O. pannosa* subsp. *cardiophylla* (*F_ST_* = 0.12–0.68). Notably, the small and isolated Kangaroo Island collection locality at Kingscote (KIN), had disproportionately higher levels of *F_ST_* compared to all other collection localities (*F_ST_* = 0.36–0.68). Collection localities within the Fleurieu Peninsula and southeast SA, show moderate differentiation (*F_ST_* = 0.17–0.22) but are highly differentiated from the geographically distant and isolated Victorian collection localities (*F_ST_* = 0.36–0.59). The collection localities within the Victorian region showed moderate genetic differentiation among Anglesea (ANG) and Brisbane Ranges (BR) (*F_ST_* = 0.20) but high when compared to the most isolated and northern Victorian locality Rushworth (RUS) (*F_ST_* = 0.46 and 0.42 respectively).

### 3.2. Genetic Diversity, Kinship, and Inbreeding Analysis

Across the dataset, the majority of genetic variation was found within individuals, but that a significant proportion of variation was also distributed between the three genetic groups (AMOVA; 42.7% and 29.9% respectively; Table 1), however there were differences between subspecies. Within *O. pannosa* subsp. *pannosa*, most genetic variation was found within individuals (67.8%) and only 11.2% between collection localities (Table 1). However, within *O. pannosa* subsp. *cardiophylla* a high proportion of the genetic variation was distributed between collection localities (43.2%; Table 1). 

**Figure 4:**
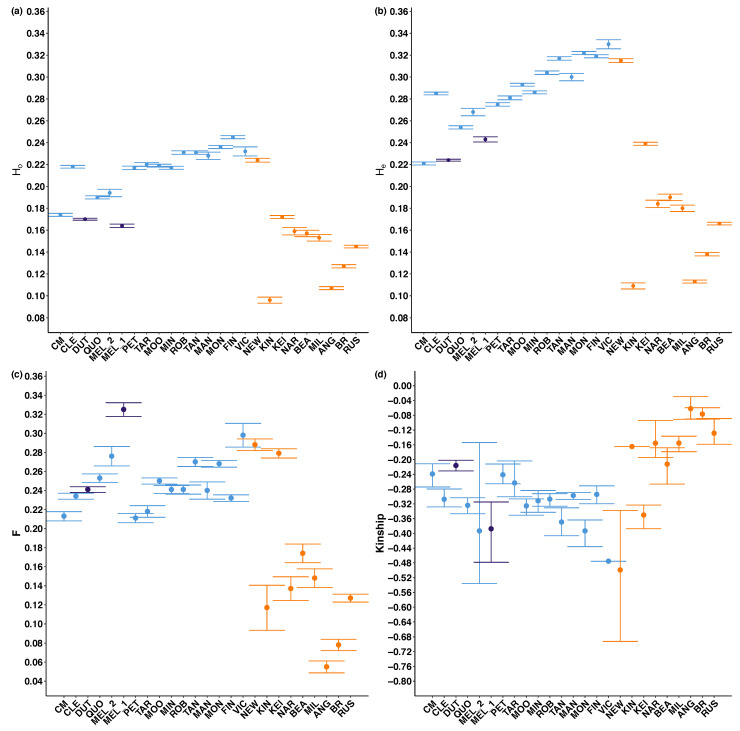
Scatterplots of (**a**) observed heterozygosity (H_O_), (**b**) expected heterozygosity (H_E_) (both vary from 0 to 1, with 0 indicating no heterozygous sites), (**c**) inbreeding coefficient (*F*) (varies from 0 to 1 and values of 0 indicate no evidence of inbreeding), and (**d**) kinship (0.5 indicates individuals are genetically identical). Bars represent 95% confidence intervals. Colours represent genetic group (blue with collection localities predominantly identified as *O. pannosa* subsp. *pannosa*, purple for *Olearia* (FR), and orange for *O. pannosa* subsp. *cardiophylla*). See Table S2 for full output. For collection locality full names refer to Table A1. For results of the Tukey pairwise comparison refer to Table S5.

Across all samples and groupings, observed heterozygosity (H_O_ = 0.096–0.245) was consistently lower than expected heterozygosity (H_E_ = 0.109–0.330; Figure 4, Table S2), and positive inbreeding coefficients (*F*) were found in all localities (*F* = 0.055–0.325; Figure 4, Table A2). Kinship was negative in each locality (kinship = −0.062 – −0.499; Figure 4, Table A2).

Within *Olearia* (FR), genetic diversity was low (H_O_ = 0.164–0.170; Figure 4, Table A2), historical inbreeding was high (*F* = 0.241–0.332; Figure 4, Table A2), but levels of kinship were low to moderate (kinship = −0.388 – −0.216; Figure 4, Table A2). *Olearia* (FR) samples collected at Melrose (MEL_1) had highest inbreeding (*F* = 0.332) but lower levels of kinship (kinship = −0.388). Within *O. pannosa* subsp. *pannosa,* levels of genetic diversity (H_O_ = 0.174–0.245; Figure 4, Table A2) and inbreeding (*F* = 0.211–0.330; Figure 4, Table A2) did not vary much between localities. There was greater variation in kinship (kinship = −0.476–−0.242; Figure 4, Table A2), with the admixed locality Victor Harbor (Vic) having the lowest levels of kinship (kinship = −0.476; Figure 4, Table A2). There is a pattern of lower kinship in the localities with higher inbreeding. There is also a general trend of expected heterozygosity (H_E_) and observed heterozygosity (H_O_) increasing the closer collection localities are to the Fleurieu Peninsula in southeast South Australia, align with increased levels of admixture detected in this region (Figure 4, Table A2). Within *O. pannosa* subsp. *cardiophylla,* Newland Head (NEW) and Keith (KEI) had the highest levels of genetic diversity and inbreeding (H_E_ = 0.315 and 0.239, H_O_ = 0.224 and 0.172, *F* = 0.288 and 0.279, respectively; Figure 4, Table A2), and lowest levels of kinship (kinship = −0499 and −0.350, respectively; Figure 4, Table A2). All other localities had moderate expected heterozygosity (H_E_ = 0.09–0.190; Figure 4, Table A2). lower inbreeding coefficients than *O. pannosa* subsp. *pannosa* (*F* = 0.08–0.17; Figure 4, Table A2), but high levels of kinship (kinship = −0.213 – −0.062; Figure 4, Table A2). Furthermore, *O. pannosa* subsp. *cardiophylla* was the only group to have highly related individuals removed; Brisbane Ranges (BR), Kangaroo Island (KIN), and Anglesea (ANG). 

The ANOVA analysis and Tukey pairwise comparisons of observed heterozygosity (H_O_), expected heterozygosity (H_E_), inbreeding (*F*), and kinship, revealed significant differences for *O. pannosa* subsp. *pannosa* and *O. pannosa* subsp. *cardiophylla* (*p* < 0.001; Table A5). There was no significant difference in Ho, He, F, and kinship for *Olearia* (FR) compared to *O. pannosa* subsp. *pannosa* (*p* > 0.05; Table A5). The only significant difference between *Olearia* (FR) and *O. pannosa* subsp. *cardiophylla* was inbreeding coefficient (*F*) in *Olearia* (FR) (*p* = 0.02; Table A5).

### 3.3. Leaf Trait Analysis

**Figure 5:**
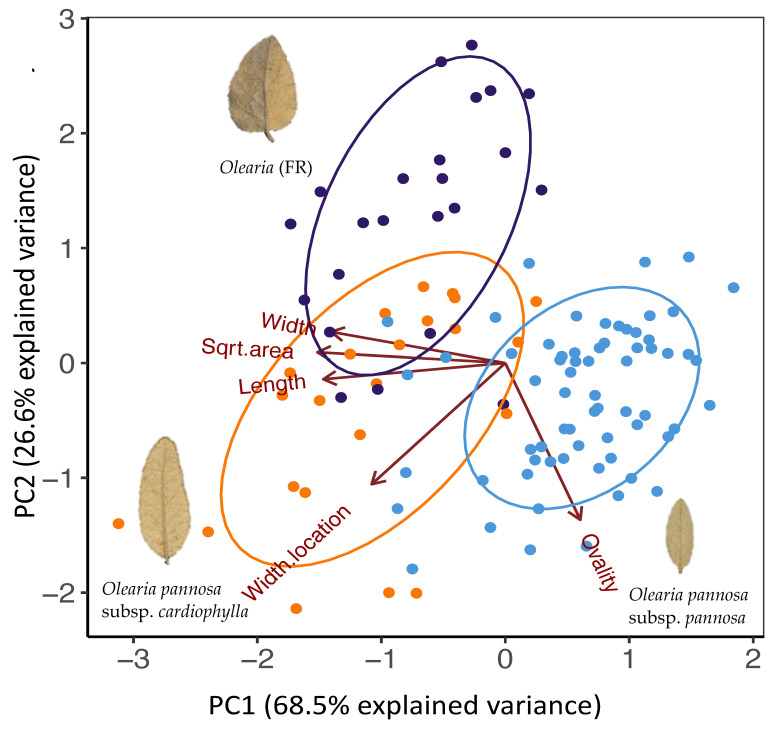
PCA of leaf shape variation. Colours represent genetic cluster assignment identified in the ADMIXTURE and DAPC analyses (blue: collection localities identified as *O. pannosa* subsp. *pannosa* group, purple: *Olearia* (FR), orange: *O. pannosa* subsp. *cardiophylla*). Images of leaves represent the typical shape found in non-admixed populations of each genetic cluster. The *O. pannosa* subsp. *cardiophylla* cluster leaf is from Naracoorte (NAR), the *Olearia* (FR) cluster from (MEL), and the *O. pannosa* subsp. *pannosa* cluster leaf is also from Melrose (MEL). Note that as the site Melrose (MEL) contains individuals belonging different genetic clusters. Those belonging to *Olearia* (FR) have been labelled as MEL_1 and *O. pannosa.* subsp. *pannosa* have been labelled as MEL_2.

Principal component analysis of all leaf measurements revealed clear clustering of samples, which broadly aligned with the three genetic groups, but with some overlap, indicating morphological intergrades (Figure 5). Principal component 1 (PC1) explained 68.5% of the variance and mainly explained by variation in length, width, and surface area of leaves (Figure A5). *Olearia pannosa* subsp. *pannosa* separated from all other samples on axis 1 due to their shorter, smaller leaves. Principal component 2 (PC2) explained 25.6% of the variance and mainly explained variation in leaf ovality and location of the widest point of the leaf. On this axis, the majority of *Olearia* (FR) samples were separated from *O. pannosa* subsp. *cardiophylla* and *O. pannosa* subsp. *pannosa* samples due to their wider, more heart-shaped leaves. For PC1 (width, length, and leaf surface area), our ANOVA analysis found significant differences between *O. pannosa* subsp. *pannosa* and *Olearia* (FR)/*O. pannosa* subsp. *cardiophylla* (*p* < 0.001; Table A6). For PC2 (ovality and location of the widest point of the leaf), differences between *O. pannosa* subsp. *pannosa* and *O. pannosa* subsp. *cardiophylla* were not significant, but both groups differed significantly to *Olearia* (FR) (*p* < 0.001; Table A6).

### 3.4. Environmental Associations

**Figure A4:**
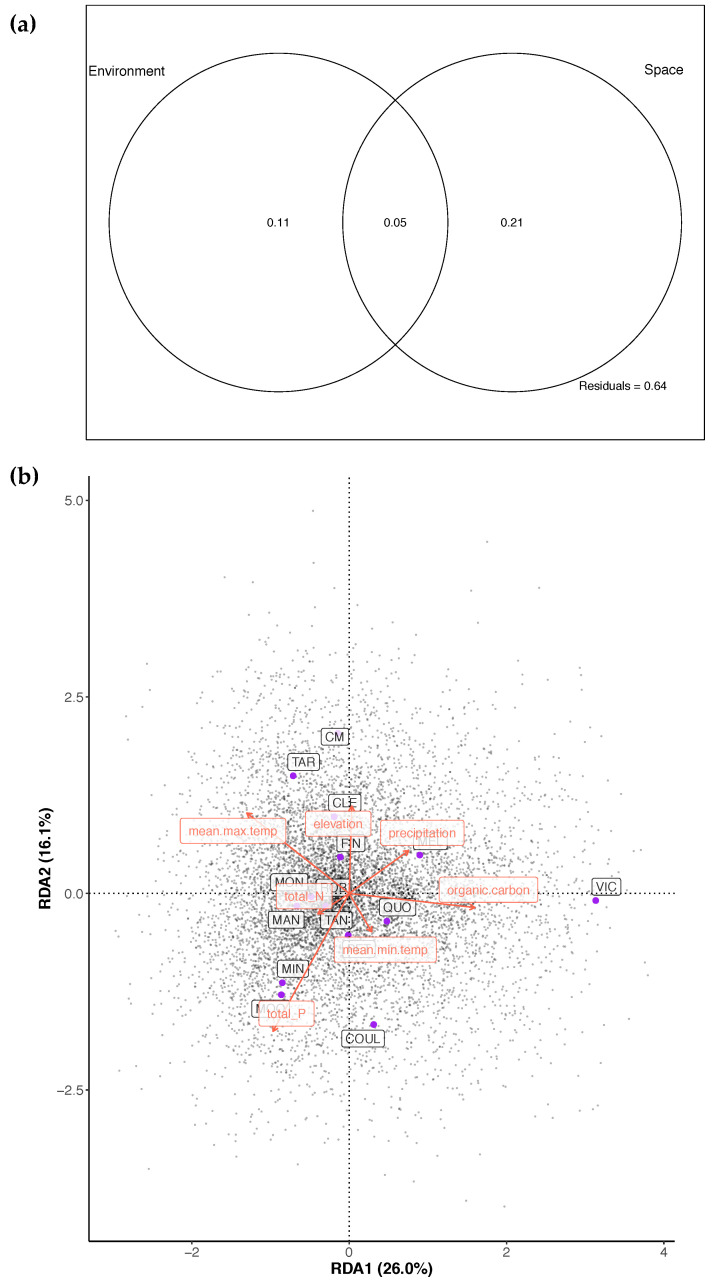
(**a**) Venn diagram showing results from variation partitioning, with environmental variables and spatial distribution used as explanatory variables, showing that 11% of the genetic variance is uniquely explained by environment. (**b**) Triplot for the first two axis of a partial redundancy analysis with environment as explanatory variables conditioned on spatial distribution.

The redundancy analysis of *O. pannosa* subsp. *pannosa* collection localities revealed that spatial distribution explained a significant proportion of the variance in allele frequencies (Table 2, Figure A4). The partial redundancy analysis, which looked at the proportion of variance explained by the seven environmental variables (mean annual maximum temperature (°C), mean annual minimum temperature (°C), annual precipitation (mm), total nitrogen (%), total phosphorus (%), organic carbon (%), and elevation (m)) after conditioning on spatial distribution, revealed that 11% of the variance in allele frequencies is significantly explained by the included environmental factors (Table 2, Figure A4). Forward selection of the environmental variables retained only mean maximum temperature as a significant contributor to the model, suggesting that this environmental variable associates with genetic variation.

The redundancy analysis of leaf shape within *O. pannosa* subsp. *pannosa* samples included the same seven environmental variables as for the allele frequency RDA. When running the RDA with only spatial distribution as the explanatory variable, 13% of the variance in leaf shape was significantly explained Table 2). With environment as the explanatory variable conditioned on spatial distribution only 3% of the variance is explained and was non-significant (Table 2). When partitioning the variance in leaf measures among the spatial and environmental variables, 17% of the variation is explained by the combination of both environment and spatial distribution. The environmental variables (particularly temperature and precipitation) do co-vary with spatial distribution, so it is difficult to tease apart the effects of the environment on leaf shape (i.e., environmentally determined) from differences in leaf shape that may be due to demography. However, as for the genotype RDA, the forward selection process revealed maximum annual temperature, as well as total nitrogen, as significant contributors to the model, once again suggesting that temperature associates with variation among these populations.

### 3.5. Habitat Suitability Modelling

**Figure A5:**
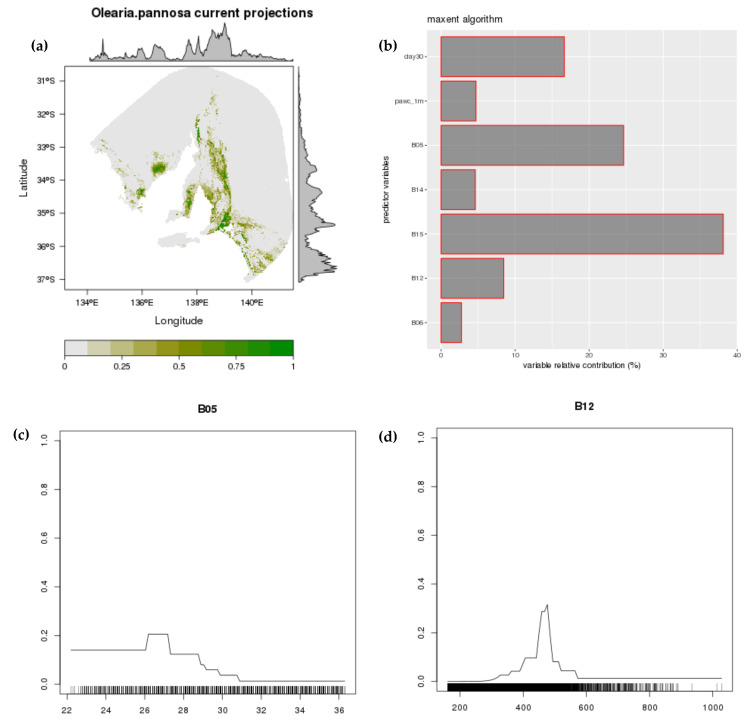
Projected distribution of *O. pannosa* subsp. *pannosa* (MaxEnt). Current distribution (**a**); variable relative contribution (**b**); two example response curves (mean maximum temperature of the warmest month; (**c**) and mean annual rainfall; (**d**)).

**Figure A6:**
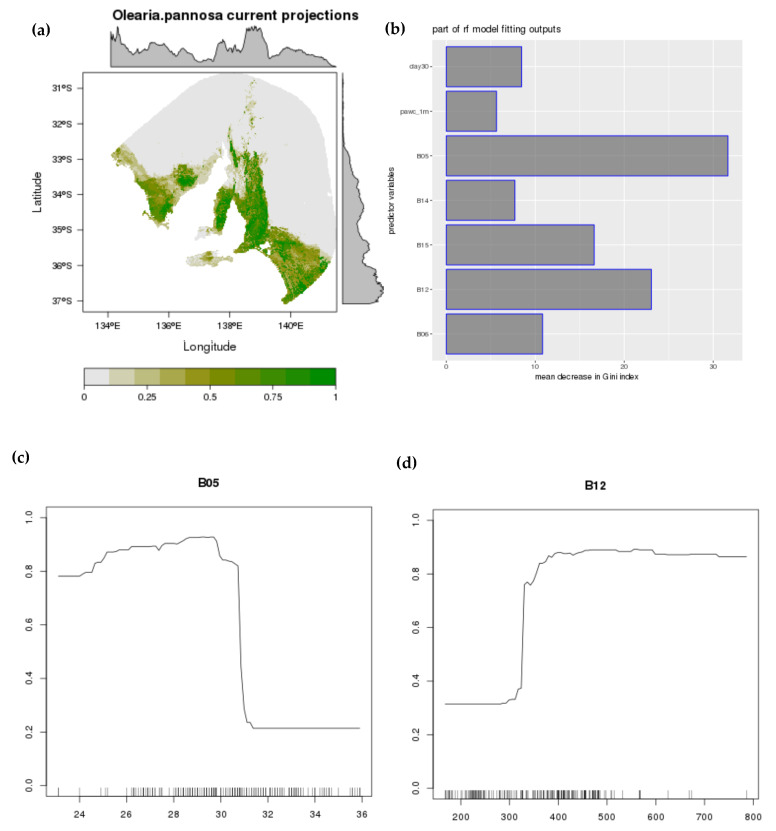
Projected distribution of *O. pannosa* subsp. *pannosa* (Random Forest). Current distribution (**a**); variable importance (**b**); two example response curves (mean maximum temperature of the warmest month; (**c**) and mean annual rainfall; (**d**)).

Final models were fitted with two edaphic (% soil clay—clay30; % available water capacity—pawc1m), two temperature predictors (mean minimum temperature of the coldest month—bio6; mean maximum temperature of the warmest month—bio5), and three rainfall (mean annual rainfall—bio12; mean rainfall of the driest month—bio14; precipitation seasonality/coefficient of variation—bio15) predictors (Figure A5 and Figure A6). Distribution models showed *O. pannosa* subsp. *pannosa* occurs within a consistent climatic band intermediate between the mesic areas and the more arid inland. Peak suitability was associated with mean annual rainfall of 400–500 mm (occupied niche 330–670 mm) and mean summer temperature maxima <29 °C (MaxEnt; <30 °C for Random Forest; occupied niche 25–31 °C). For MaxEnt, precipitation seasonality had the highest variable relative contribution followed by mean maximum temperature of the warmest month. For random forest, mean maximum temperature of the warmest month had the highest variable importance followed by mean annual rainfall.

## 4. Discussion

Our analysis of genomic and leaf shape variation in the threatened daisy bush, *Olearia pannosa,* unearthed a complex picture that is not in agreement with the current subspecific classifications for the species. A key finding of our study was the identification of a likely cryptic species *Olearia* (FR) in the Flinders Ranges which exists only in small, fragmented collection localities. For the two subspecies, *O. pannosa* subsp. *pannosa* and *O. pannosa* subsp. *cardiophylla*, we identified a strong east/west genetic divide with signatures of admixture between the two subspecies and substructure within them. Genetic diversity was reduced in *Olearia* (FR) compared to *O. pannosa* subsp. *pannosa*, and was substantially lower in the small, isolated localities of *O. pannosa* subsp. *cardiophylla*. Our analyses of associations between environmental and genetic and phenotypic variation identified a strong association with maximum temperature. This finding was further supported by habitat suitability modelling, which identified maximum temperature and precipitation as key variables in determining species’ distribution. Our results have implications for the conservation management for *O. pannosa*, particularly for the newly identified genetic group *Olearia* (FR) in the Flinders Ranges.

### 4.1. Genetic Structure, Diversity, Inbreeding, and Kinship 

We found evidence of strong differentiation among collection localities, with clear support for three major genetic groups across all analyses and substructure with them. Previously, *O. pannosa* plants found in the southern Flinders Ranges were thought to be the same subspecies as *O. pannosa* subsp. *cardiophylla* found in southeastern South Australia and Victoria. However, our analysis reveals that these southern Flinders Ranges plants (*Olearia* (FR)), sampled at Dutchman’s Stern (DUT) and Melrose (MEL), are not only strikingly distinct genetically from their neighbouring collection localities of *O. pannosa* subsp. *pannosa* but also from all other collection localities of *O. pannosa* subsp. *cardiophylla* in the southeastern South Australia and Victoria. This is in clear contrast to the current classification based on morphology, where *O. pannosa* in the Flinders Ranges is considered to be part of the same subspecies as *O. pannosa* subsp. *cardiophylla* found in Victoria. The apparent lack of gene flow among *Olearia* (FR) and neighbouring *O. pannosa* subsp. *pannosa* collection localities strongly suggests that there are reproductive barriers present between these two clusters and these collection localities likely represent two distinct species. Notably, both *Olearia* (FR) collection localities have high genetic differentiation between them, low genetic diversity, and high historical inbreeding. These findings are consistent with results from other recent genomic studies of threatened and endangered species [67,68,69]. As far as we know, this is a new finding, signalling the need for taxonomic description of this newly discovered genetic group and a conservation management strategy to be developed to ensure its survival. 

The remaining samples could be assigned to either the *O. pannosa* subsp. *pannosa* or *O. pannosa* subsp. *cardiophylla* genetic groups, which are split by an east to west genetic divide. These two genetic groups differed significantly in levels of genetic diversity, inbreeding and kinship, with *O. pannosa* subsp. *cardiophylla* having lower levels of genetic diversity and inbreeding but higher levels of kinship. Interestingly, a self-compatibility test was undertaken at the Brisbane Ranges locality which found that individuals could self-pollinate and set seed, and inbreeding was flagged as a concern in *O. pannosa* subsp. *cardiophylla* [34]. Certainly *O. pannosa* subsp. *cardiophylla* shows signs of population genetic decline indicated by the low genetic diversity and high kinship, and our findings consistent with the fact that the Victorian populations are now small and demographically isolated. There is also potential for elevated levels of clonality in these collection localities [70,71], but as our sampling protocol was designed to minimise the likely number of clones in our dataset, it is not possible for us to calculate the proportion of clones within collection localities. We did, however, detect one likely clone in the Kangaroo Island (KIN) collection locality, which also had the lowest levels of genetic diversity. Nevertheless, due to the limitations in our study, we advise further research to test mating system dynamics.

We observed genetic admixture on the southern Fleurieu Peninsula and southeastern SA, with a shift from a collection locality with high assignment to the *O. pannosa* subsp. *pannosa* group at Victor Harbor (VIC) to a collection locality with high assignment to the *O. pannosa* subsp. *cardiophylla* group at the closely located (~12 km) collection locality at Newland Head (NEW). Interestingly, Newland Head (NEW) had the variation in pairwise kinship between individuals within *O. pannosa* subsp. *cardiophylla.* Genetic admixture patterns were reflected in the leaf shape analysis, with intermediate morphologies described at these sites. Distribution records for both subspecies do not align with these findings, likely due to the difficulties with identification in these admixed sites, highlighting the intrinsic difficulty of defining subspecies boundaries. There was further substructure within *O. pannosa* subsp. *cardiophylla*, which is represented by several isolated and geographically distant collection localities. Our findings in Victoria are in agreement with the results of Smith, James and Ladiges [34] who also found that the collection site Rushworth (RUS) was genetically differentiated from the Brisbane Ranges (BR) and Anglesea (ANG) sites which could be explained by the large geographic distance between these localities (~150 km and ~220 km respectively).

Overall, the genetic structure results reflect the life history traits and disjunct range of *O. pannosa* as gene flow will be limited, resulting in greater genetic drift. Therefore, the strong differentiation in isolated collection localities is unsurprising. Similar patterns have been found in other threatened species [67,68]. For example, genomic analysis of the endangered Australian daisy (*Rutidosis leptorrhynchoides*) found strong genetic differentiation between geographically isolated collection localities [69].

### 4.2. Leaf Shape and Taxonomy 

Our leaf shape analysis showed that the three main genetic clusters display relatively distinct leaf morphologies. Specimens measured from the *O. pannosa* subsp. *pannosa* genetic cluster had smaller, more oval shaped leaves compared to plants belonging to the *Olearia* (FR) and *O. pannosa* subsp. *cardiophylla* genetic groups, which we found to overlap more in size than shape. Previous taxonomic work on the species has classified subspecies by morphological traits and demonstrated clear differences between the leaf shapes of *O. pannosa* subsp. *pannosa* in South Australia and *O. pannosa* subsp. *cardiophylla* in Victoria [34], similar to what we find here. However, this previous analysis showed that *Olearia* (FR) samples (previously *O. pannosa* subsp. *cardiophylla* in South Australia) clustered with *O. pannosa* subsp. *cardiophylla* samples from Anglesea (ANG), Victoria. Here, we show that whilst the leaf shape of *Olearia* (FR) is more similar to *O. pannosa* subsp. *cardiophylla* than to *O. pannosa* subsp. *pannosa*, there are differences between them, with *Olearia* (FR) having more heart-shaped leaves. Geographic distance between *Olearia* (FR) and *O. pannosa* subsp. *cardiophylla* is extremely large (~315 km between Melrose (MEL) and the closest *O. pannosa* subsp. *cardiophylla* locality at Newland Head (NEW)). We also show that genetic divergence is high between *Olearia* (FR) and all other *O. pannosa* collection localities, suggesting they may be a distinct species, although reproductive isolation is yet to be tested. 

We observed evidence for admixture among *O. pannosa* subsp. *pannosa* and *O. pannosa* subsp. *cardiophylla* on the Fleurieu Peninsula and in parts of the Southeast of SA, indicating that gene flow is possible between these groups. If Olearia (FR) and *O. pannosa* subsp. *pannosa* were not reproductively isolated, then the strong signal of genetic structure we observe between such geographically close collection localities inhabiting very similar environments is difficult to explain. The advance in sequencing technologies in recent years has led to numerous studies that have unearthed previously unrecognised ‘cryptic species’ [71,72,73,74,75,76,77,78,79]. These are genetically distinct taxa that are classified as the same species due to morphological similarity [80]. This species complex has undergone several taxonomic revisions since its first description by Hooker in 1851 (see Smith, James and Ladiges [34] and references therein), reflecting the large amount of phenotypic variation within the species. These results are also consistent with the ongoing difficulties with taxonomy and phylogeny in the *Olearia* genus [29,30,31,32,33]. Our findings strongly suggest a cryptic species complex in *O. pannosa*. For effective threatened species management plans, a firm understanding of likely species or subspecies boundaries is essential, and our results highlight the importance of combining both genetic and morphological results methods for this approach. 

### 4.3. Environment Likely Shapes Genotype, Phenotype, and Distribution

We conducted further analyses to identify the effect of the environment across the genotype, phenotype, and distribution of *O. pannosa* subsp. *pannosa.* These analyses identified maximum temperature as a key environmental variable that is a likely selective pressure on this group. Our analyses of genetic and leaf shape variation both identified maximum temperature as explaining a proportion of the measured variation. These findings are consistent with a number of studies that have found genetic signals [81,82,83,84,85] and morphological variation [86,87,88,89,90] associated with temperature being an agent of selection on plant species. Furthermore, the current distribution of *O. pannosa* subsp. *pannosa* appears to be closely related to climate. Our climate suitability models indicated specific constraints with respect to mean annual rainfall and summer maximum temperature gradients, with the species occurring within a transition zone between the more mesic and arid zones of the surrounding region [91]. Many collection localities of *O. pannosa* subsp. *pannosa* sit at the warm/arid thresholds of these key climatic gradients beyond which modelled probability of occurrence drops off markedly. 

Taken together these results suggest that the current climatic trends towards higher summer maxima and increasing aridity are likely to have a detrimental impact on many collection localities. However, the few localities in cooler, wetter habitats may, in fact, benefit in the short term from climate change, leading to shifts in distribution and abundance. Combined, these results highlight the potential vulnerability of *O. pannosa* subsp. *pannosa* to the imminent effects of climate change and should be considered when planning conservation strategies for the species.

### 4.4. Conservation Importance and Implications for Each Genetic Group 

#### 4.4.1. *Olearia pannosa* (FR)

*Olearia pannosa* (FR) had low levels of genetic diversity across both collection localities and the collection locality MEL (Melrose) had the highest level of inbreeding across the whole dataset. We also found moderately high genetic differentiation between the two collection localities Dutchman’s Stern (DUT) and Melrose (MEL). Concerningly, as there are only a few known stands of these individuals, all with small census estimates, this suggests an endangered species, or at least a subspecies which contains distinct genetic variation that will be lost if these they are not conserved. An immediate taxonomic review is essential to address these issues. We suggest a multi-level management strategy centred for this group. Firstly, we recommend in-situ threat abatement (i.e., protection from grazing pressure) and natural recruitment (i.e., creating disturbance to encourage recovery from the seedbank) [92]. We also recommend developing a strategy to aid the recovery of genetic diversity through a translocation plan which facilitates gene flow between the two isolated stands [92]. This could be developed through the ex-situ establishment of a managed seed production orchard to provide high quality seed [93]. Furthermore, further research could be informative, such as whole genome sequencing and breeding experiments, to ascertain (a) whether *Olearia* (FR) truly is a distinct, isolated species from *O. pannosa* and (b) what the functional genomic differences are between these divergent taxa.

#### 4.4.2. *Olearia pannosa* subsp. *pannosa*

Within *O. pannosa* subsp. *pannosa*, there is evidence of some degree of historical gene flow across collection localities and low population substructure. This genetic group had higher levels of genetic diversity compared to *O. pannosa* subsp. *cardiophylla.* Observed heterozygosity was consistently lower than expected heterozygosity and was reflected in the significantly higher levels of historical inbreeding in *O. pannosa* subsp. *pannosa* than the other two genetic groups. Inbreeding has previously been flagged as a concern for *O. pannosa* [34] and historical selfing in this group may be one explanation for the results. Due to the substructure that we found between isolated collection localities on the Eyre Peninsula (Coulta (COUL) Cummins (CM) and Cleeves (CLE)) and the rest of the group, we recommend that management efforts be focused here. Facilitating gene flow between these stands through translocations or increasing connectivity between may be advantageous.

As our results identify maximum temperature as potential agent of selection for this group, stands in more arid regions may experience detrimental effects from climate change. We recommend close monitoring, particularly of the more northern and arid localities. Apart from collection localities on the Eyre Peninsula, there is evidence of gene flow and higher levels of genetic diversity compared to other genetic groups. For these stands, we recommend a management approach which maintains connectivity and gene flow among localities. However, the potential future effect of aridification on this genetic group should be considered in future management strategies, and a climate-adjusted seed sourcing strategy [18] could be implemented (i.e., introducing seed from more arid areas to southern stands to bolster their adaptive capacity to arid conditions). 

#### 4.4.3. *Olearia pannosa* subsp. *cardiophylla*

We found that *O. pannosa* subsp. *cardiophylla* had the lowest levels of both historical inbreeding and genetic diversity, yet the highest levels of kinship. We also found that three of the four sites which had lowest genetic diversity and inbreeding in this group were the only collection sites that had to have highly related individuals removed across the whole dataset. There was considerable substructure in this genetic group, particularly between sites in Southeast SA and Victoria, and between sites within Victoria. It is likely that the large range disjunction of this group is driving genetic drift and genetic divergence. To develop an appropriate conservation management plan for *O. pannosa* subsp. *cardiophylla*, there are several knowledge gaps which still need to be addressed, particularly around the genetic divergence we observed between *O. pannosa* subsp. *cardiophylla* and the two other genetic groups, and the consequences that the high levels of substructure we found within this genetic group may have on mixing seed between localities (e.g., outbreeding depression). We suggest the establishment of a common garden trial which explores the effects on fitness and genetic diversity of genetic mixing between localities within this genetic group and amongst the three genetic groups. Until this information is known, in a scenario with high structure but low genetic diversity and inbreeding, Ottewell, Bickerton, Byrne, and Lowe [88] suggests a cautious management approach to minimise the risk of outbreeding depression with a focus on in-situ recovery of genetic diversity through the seed bank and a composite translocation strategy [19].

## 5. Conclusions

In summary, combining range-wide genomic and leaf shape analysis of the threatened Australian daisy *Olearia pannosa* revealed a likely cryptic species (*Olearia* (FR)) and identified the need to redefine the geographic intergrade between existing subspecific boundaries (*O. pannosa* subsp. *cardiophylla* and *O. pannosa* subsp. pannosa). Levels of genetic diversity and inbreeding differed between the three genetic groups, suggesting each requires a separate management strategy which we define below.

- *Olearia* (FR)–We suggest a management strategy centred around in-situ threat abatement and disturbance to encourage seedbank recovery, the recovery of genetic diversity through a translocation plan to encourage gene flow between the two isolated stands, and the development of a seed production orchard to supply the seed.
- *O. pannosa* subsp. *pannosa*—The main priority is to facilitate gene flow between existing stands or to increase connectivity between them, especially in an arid-to-mesic direction since it appears that maximum temperature is an important agent of selection.
- *O. pannosa* subsp. *cardiophylla*—It is likely that the large range disjunction of this group is driving genetic divergence. To develop an appropriate conservation management plan, several knowledge gaps still need to be addressed, particularly to assess the potential for outbreeding depression. Until then, we recommend priority is given to in-situ recovery of genetic diversity.

## Figures and Tables

**Table 1:** Nested analysis of molecular variance (AMOVA) analysing the distribution of genetic diversity (**a**) across all collection localities, (**b**) within *O. pannosa* subsp. *pannosa,* and (**c**) within *O. pannosa* subsp. *cardiophylla*. Including standard deviations (SD; obtained through jackknifing over loci) and 95% confidence intervals (c.i. 95%; obtained through bootstrapping over loci) of F statistics.

**(a) All collection localities**							
Source of variation	Nested in	%var	F	Std.Dev.	c.i.2.5%	c.i.97.5%	*p*
Within individual	--	42.7	0.573	0.002	0.569	0.578	--
Among individuals	Collection locality	11.2	0.208	0.003	0.203	0.213	<0.001
Among collection localities	group	16.2	0.231	0.001	0.228	0.234	<0.001
Among group	--	29.9	0.299	0.002	0.294	0.304	<0.001
**(b) *Olearia pannosa* subsp. *pannosa* only**							
Source of variation	Nested in	%var	F	Std.Dev.	c.i.2.5%	c.i.97.5%	*p*
Within individual	--	67.8	0.322	0.002	0.317	0.327	--
Among individuals	Collection locality	21.3	0.239	0.003	0.234	0.244	<0.001
Among collection localities	--	10.9	0.109	0.001	0.107	0.111	<0.001
**(c) *Olearia pannosa* subsp. *cardiophylla* only**							
Source of variation	Nested in	%var	F	Std.Dev.	c.i.2.5%	c.i.97.5%	*p*
Within individual	--	48.2	0.518	0.003	0.512	0.525	--
Among individuals	Collection locality	8.6	0.152	0.004	0.144	0.16	<0.001
Among collection localities	--	43.2	0.432	0.003	0.427	0.438	<0.001

**Table 2:** Results from partial redundancy analyses. Significance was assessed using an ANOVA-like permutation test with 1000 permutations.

Response Variable	Explanatory Variables	Condition Variables	r^2^	F	*p*
Allele frequencies	Space	-	0.36	2.59	<0.001
	Environment	Space	0.11	1.64	0.038
Leaf shape	Space	-	0.13	5.12	0.002
	Environment	Space	0.03	2.13	0.086

## Data Availability

Reads and mapping files will be archived at the NCBI SRA (accession number TBA).

## Supplementary Materials

The following are available online at https://www.mdpi.com/article/10.3390/life11060553/s1, Supplementary File S1—The variant call format (VCF) file will be made available as supplementary (Olearia_pannosa.vcf). Reads and mapping files will be archived at the NCBI SRA (accession number TBA). Supplementary File S2—the covariates from the redundancy analysis (genetic_RDA_covariates.xlsx).

## Appendix A

**Table A1:** Summary of region, collection locality, collection ID, collection site coordinates, locality voucher identification (provided by the State Herbarium of South Australia, locality genetic group assignment, and the 2018 census estimate for each collection locality

Region	Collection Locality	Collection ID	Latitude	Longitude	Subspecies (SA Herbarium ID)	Genetic Group	2018 Census Estimate
Eyre Peninsula	Coulta	COUL	−34.38945	135.4347	*O. pannosa* subsp. *pannosa*	*O. pannosa* subsp. *pannosa*	1
	Cummins	CM	−34.32097	135.9643	*O. pannosa* subsp. *pannosa*	*O. pannosa* subsp. *pannosa*	440
	Cleeve	CLE	−33.6998	136.5024	*O. pannosa* subsp. *pannosa*	*O. pannosa* subsp. *pannosa*	490
Flinders Ranges	Dutchman’s Stern	DUT	−32.30784	137.9728	*O. pannosa* subsp. *cardiophylla*	*O. pannosa* (FR)	400
	Quorn	QUO	−32.49042	138.0478	*O. pannosa* subsp. *pannosa*	*O. pannosa* subsp. *pannosa*	6300
	Melrose	MEL	−32.69477	138.1048	*O. pannosa* subsp. *pannosa*	*O. pannosa* subsp. *pannosa*	231
	Melrose	MEL	−32.71896	138.0935	*O. pannosa* subsp. *cardiophylla*	*O. pannosa* (FR)	345
	Peterborough	PET	−32.78896	138.8835	*O. pannosa* subsp. *pannosa*	*O. pannosa* subsp. *pannosa*	60
	Tarcowie	TAR	−34.65343	138.8208	*O. pannosa* subsp. *pannosa*	*O. pannosa* subsp. *pannosa*	29
Yorke Peninsula	Moonta	MOO	−34.54376	137.7825	*O. pannosa* subsp. *pannosa*	*O. pannosa* subsp. *pannosa*	370
	Minlaton	MIN	−34.6748	137.7199	*O. pannosa* subsp. *pannosa*	*O. pannosa* subsp. *pannosa*	385
Clare Valley/Barossa	Robertstown	ROB	−33.85449	139.0413	*O. pannosa* subsp. *pannosa*	*O. pannosa* subsp. *pannosa*	1523
	Tanunda	TAN	−34.38884	138.8028	*O. pannosa* subsp. *pannosa*	*O. pannosa* subsp. *pannosa*	368
Murray River	Mannum	MAN	−34.8747	139.2146	*O. pannosa* subsp. *pannosa*	*O. pannosa* subsp. *pannosa*	22
	Monarto	MON	−35.22497	139.0047	*O. pannosa* subsp. *pannosa*	*O. pannosa* subsp. *pannosa*	301
Fleurieu Peninsula	Finniss	FIN	−35.31793	138.8836	*O. pannosa* subsp. *pannosa*	*O. pannosa* subsp. *pannosa*	513
	Victor Harbor	VIC	−35.47631	138.7684	*O. pannosa* cf. subsp. *cardiophylla*	*O. pannosa* subsp. *pannosa*	115
	Newland Head	NEW	−35.61022	138.5018	*O. pannosa* cf. subsp. *cardiophylla*	*O. pannosa* subsp. *cardiophylla*	31
Kangaroo Island	Kingscote	KIN	−35.57406	137.576	*O. pannosa* cf. subsp. *cardiophylla*	*O. pannosa* subsp. *cardiophylla*	12
Southeast SA	Keith	KEI	−36.14328	140.4094	*O. pannosa* cf. subsp. *cardiophylla*	*O. pannosa* subsp. *cardiophylla*	514
	Naracoorte	NAR	−37.11145	140.5361	*O. pannosa* cf. subsp. *cardiophylla*	*O. pannosa* subsp. *cardiophylla*	80
	Beachport	BEA	−37.28313	139.9409	*O. pannosa* cf. subsp. *cardiophylla*	*O. pannosa* subsp. *cardiophylla*	14
	Millicent	MIL	−37.68687	140.5147	*O. pannosa* cf. subsp. *cardiophylla*	*O. pannosa* subsp. *cardiophylla*	18
Victoria	Anglesea	ANG	−38.37622	144.245	*O. pannosa* cf. subsp. *cardiophylla*	*O. pannosa* subsp. *cardiophylla*	Unknown
	Brisbane Ranges	BR	−37.90723	144.232	*O. pannosa* cf. subsp. *cardiophylla*	*O. pannosa* subsp. *cardiophylla*	Unknown
	Rushworth	RUS	−36.62514	145.1126	*O. pannosa* cf. subsp. *cardiophylla*	*O. pannosa* subsp. *cardiophylla*	Unknown

**Table A2:** Site ID, genetic group, highly related individuals removed from collection locality, sample size after filtering (n), number of variant sites after filtering for a minor allele count of 1, observed (HO) and expected (HE) heterozygosity (both vary from 0 to 1, with 0 indicating no diversity), inbreeding coefficient (*F*) (varies from 0 to 1 and values of 0 indicate no evidence of inbreeding), and Kinship (0.5 indicates samples are genetically identical. The lower the kinship coefficient, the lower the level of kinship between individuals).

Collection ID	Genetic Group	Highly Related IndividualsRemoved	n	Variant Sites	H_O_	H_E_	F	Kinship
COUL	*O. pannosa* subsp. *pannosa*	-	1	1379	---	---	---	---
CM	*O. pannosa* subsp. *pannosa*	-	8	5635	0.174 0.173 to 0.175)	0.221 (0.220 to 0.222)	0.213 (0.208 to 0.218)	−0.239 (−0.274 to −0.211)
CLE	*O. pannosa* subsp. *pannosa*	-	10	7479	0.218 (0.217 to 0.219)	0.285 (0.284 to 0.286)	0.234 (0.231 to 0.237)	−0.308 (−0.328 to −0.280)
DUT	*O. pannosa* (FR)	-	21	4612	0.17 (0.169 to 0.171)	0.224 (0.223 to 0.225)	0.241 (0.238 to 0.244)	−0.216 (−0.231 to −0.202)
QUO	*O. pannosa* subsp. *pannosa*	-	7	6457	0.19 (0.189 to 0.191)	0.254 (0.253 to 0.255)	0.253 (0.249 to 0.257)	−0.324 (−0.347 to −0.303)
MEL_2	*O. pannosa* subsp. *pannosa*	-	3	4931	0.194 (0.191 to 0.197)	0.268 (0.265 to 0.271)	0.276 (0.266 to 0.286)	−0.394 (−0.536 to −0.154)
MEL_1	*O. pannosa* (FR)	-	6	3968	0.164 (0.162 to 0.166)	0.243 (0.241 to 0.245)	0.325 (0.318 to 0.332)	−0.388 (−0.478 to −0.315)
PET	*O. pannosa* subsp. *pannosa*	-	6	6666	0.217 (0.215 to 0.219)	0.275 (0.273 to 0.277)	0.211 (0.206 to 0.216)	−0.242 (−0.265 to −0.212)
TAR	*O. pannosa* subsp. *pannosa*	-	5	6468	0.22 (0.218 to 0.222)	0.281 (0.279 to 0.283)	0.218 (0.212 to 0.224)	−0.264 (−0.301 to −0.204)
MOO	*O. pannosa* subsp. *pannosa*	-	9	7613	0.219 (0.218 to 0.220)	0.293 (0.292 to 0.294)	0.250 (0.247 to 0.253)	−0.326 (−0.351 to −0.307)
MIN	*O. pannosa* subsp. *pannosa*	-	8	7254	0.217 (0.216 to 0.218)	0.286 (0.285 to 0.287)	0.241 (0.237 to 0.245)	−0.312 (−0.343 to −0.284)
ROB	*O. pannosa* subsp. *pannosa*	-	6	7337	0.231 (0.229 to 0.233)	0.304 (0.302 to 0.306)	0.241 (0.236 to 0.246)	−0.307 (−0.326 to −0.292)
TAN	*O. pannosa* subsp. *pannosa*	-	6	7645	0.231 (0.229 to 0.233)	0.317 (0.315 to 0.319)	0.270 (0.265 to 0.275)	−0.370 (−0.406 to −0.331)
MAN	*O. pannosa* subsp. *pannosa*	-	3	5571	0.228 (0.225 to 0.231)	0.300 (0.297 to 0.303)	0.240 (0.231 to 0.249)	−0.298 (−0.308 to −0.290)
MON	*O. pannosa* subsp. *pannosa*	-	8	8299	0.236 (0.235 to 0.237)	0.322 (0.321 to 0.323)	0.268 (0.265 to 0.271)	−0.394 (−0.436 to −0.364)
FIN	*O. pannosa* subsp. *pannosa*	-	8	8224	0.245 (0.244 to 0.246)	0.319 (0.318 to 0.320)	0.232 (0.229 to 0.235)	−0.295 (−0.320 to −0.271)
VIC	*O. pannosa* subsp. *cardiophylla*	-	2	4694	0.232 (0.228 to 0.236)	0.330 (0.326 to 0.334)	0.298 (0.286 to 0.310)	−0.476 (−0.476 to −0.476)
NEW	*O. pannosa* subsp. *cardiophylla*	-	5	6485	0.224 (0.222 to 0.226)	0.315 (0.313 to 0.317)	0.288 (0.282 to 0.294)	−0.499 (−0.692 to −0.338)
KIN	*O. pannosa* subsp. *cardiophylla*	1	2	1393	0.096 (0.093 to 0.099)	0.109 (0.106 to 0.112)	0.117 (0.093 to 0.141)	−0.165 (−0.165 to −0.165)
KEI	*O. pannosa* subsp. *cardiophylla*	-	8	5436	0.172 (0.171 to 0.173)	0.239 (0.238 to 0.240)	0.279 (0.274 to 0.284)	−0.350 (−0.387 to −0.323)
NAR	*O. pannosa* subsp. *cardiophylla*	-	3	7200	0.159 (0.156 to 0.162)	0.184 (0.181 to 0.187)	0.137 (0.125 to 0.149)	−0.156 (−0.194 to −0.094)
BEA	*O. pannosa* subsp. *cardiophylla*	-	4	3552	0.157 (0.154 to 0.160)	0.190 (0.187 to 0.193)	0.174 (0.164 to 0.184)	−0.213 (−0.266 to −0.168)
MIL	*O. pannosa* subsp. *cardiophylla*	-	4	3329	0.153 (0.150 to 0.156)	0.180 (0.177 to 0.183)	0.148 (0.138 to 0.158)	−0.156 (−0.179 to −0.137)
ANG	*O. pannosa* subsp. *cardiophylla*	2	8	2525	0.107 (0.106 to 0.108)	0.113 (0.112 to 0.114)	0.055 (0.049 to 0.061)	−0.062 (−0.091 to −0.029)
BR	*O. pannosa* subsp. *cardiophylla*	3	7	3081	0.127 (0.126 to 0.128)	0.138 (0.137 to 0.139)	0.078 (0.072 to 0.084)	−0.077 (−0.090 to −0.059)
RUS	*O. pannosa* subsp. *cardiophylla*	-	11	3875	0.145 (0.144 to 0.146)	0.166 (0.165 to 0.167)	0.127 (0.123 to 0.131)	−0.129 (−0.159 to −0.089)

95% confidence intervals in parentheses.

**Table A3:** Pairwise genetic differentiation (measured by FST) between collection localities (values vary from 0–1 and values of 0 indicate no evidence of differentiation)

	COUL	CM	CLE	DUT	QUO	MEL_2	MEL_1	PET	TAR	MOO	MIN	ROB	TAN	MAN	MON	FIN	VIC	NEW	KIN	KEI	NAR	BEA	MIL	ANG	BR	RUS
**COUL**	0.00																									
**CM**	0.05	0.00																								
**CLE**	0.07	0.13	0.00																							
**DUT**	0.63	0.58	0.49	0.00																						
**QUO**	0.20	0.25	0.13	0.51	0.00																					
**MEL_2**	0.19	0.27	0.13	0.52	0.07	0.00																				
**MEL_1**	0.59	0.53	0.42	0.23	0.45	0.45	0.00																			
**PET**	0.17	0.22	0.10	0.52	0.09	0.10	0.45	0.00																		
**TAR**	0.16	0.21	0.09	0.52	0.09	0.09	0.45	0.06	0.00																	
**MOO**	0.15	0.22	0.11	0.49	0.15	0.15	0.41	0.12	0.11	0.00																
**MIN**	0.17	0.23	0.12	0.51	0.17	0.16	0.43	0.13	0.12	0.00	0.00															
**ROB**	0.11	0.20	0.07	0.50	0.12	0.11	0.41	0.08	0.06	0.07	0.08	0.00														
**TAN**	0.09	0.20	0.07	0.48	0.12	0.10	0.39	0.08	0.06	0.06	0.07	0.01	0.00													
**MAN**	0.14	0.25	0.10	0.54	0.16	0.13	0.46	0.11	0.09	0.09	0.10	0.05	0.03	0.00												
**MON**	0.08	0.19	0.07	0.46	0.11	0.09	0.37	0.07	0.05	0.06	0.07	0.01	0.00	0.02	0.00											
**FIN**	0.12	0.21	0.09	0.46	0.13	0.12	0.37	0.10	0.08	0.08	0.09	0.04	0.02	0.04	0.02	0.00										
**VIC**	0.11	0.30	0.15	0.55	0.21	0.17	0.46	0.16	0.14	0.12	0.13	0.08	0.04	0.07	0.03	0.03	0.00									
**NEW**	0.29	0.36	0.26	0.52	0.31	0.29	0.43	0.28	0.26	0.22	0.23	0.20	0.16	0.19	0.15	0.12	0.09	0.00								
**KIN**	0.72	0.52	0.39	0.66	0.46	0.49	0.64	0.43	0.42	0.36	0.38	0.36	0.31	0.41	0.29	0.27	0.36	0.22	0.00							
**KEI**	0.46	0.46	0.36	0.59	0.41	0.42	0.53	0.39	0.38	0.33	0.34	0.33	0.29	0.34	0.27	0.25	0.27	0.17	0.36	0.00						
**NAR**	0.58	0.50	0.38	0.64	0.44	0.46	0.60	0.42	0.41	0.35	0.36	0.35	0.31	0.38	0.28	0.27	0.32	0.20	0.51	0.17	0.00					
**BEA**	0.57	0.50	0.39	0.63	0.45	0.48	0.59	0.43	0.42	0.36	0.37	0.36	0.33	0.40	0.30	0.28	0.34	0.20	0.47	0.16	0.12	0.00				
**MIL**	0.59	0.51	0.40	0.64	0.46	0.49	0.60	0.44	0.43	0.37	0.38	0.37	0.33	0.41	0.31	0.28	0.35	0.22	0.50	0.18	0.16	0.12	0.00			
**ANG**	0.75	0.63	0.53	0.71	0.59	0.65	0.71	0.58	0.59	0.51	0.53	0.53	0.50	0.60	0.47	0.46	0.58	0.45	0.68	0.49	0.59	0.56	0.58	0.00		
**BR**	0.70	0.60	0.50	0.69	0.56	0.61	0.68	0.55	0.55	0.49	0.50	0.50	0.47	0.56	0.44	0.43	0.53	0.41	0.63	0.45	0.55	0.52	0.54	0.20	0.00	
**RUS**	0.64	0.57	0.47	0.66	0.53	0.56	0.64	0.52	0.52	0.46	0.47	0.47	0.44	0.51	0.41	0.40	0.47	0.36	0.55	0.42	0.49	0.47	0.48	0.46	0.42	0.00

**Table A4:** Kinship values between highly related individuals removed from the dataset (0.5 indicates samples are genetically identical. The lower the kinship coefficient, the lower the level of kinship between individuals).

Collection Locality	Collection ID Individual 1	Collection Locality	Collection ID Individual 2	Kinship	Relation
KIN	KIN_03	KIN	KIN_02	0.497758	clone
BR	BR_04	BR	BR_02	0.265698	first-degree
ANG	ANG_02	ANG	ANG_01	0.254376	first-degree
ANG	ANG_05	ANG	ANG_04	0.252064	first-degree
BR	BR_09	BR	BR_05	0.241582	first-degree
RUS	RUS_06	RUS	RUS_01	0.212617	first-degree
ANG	ANG_10	ANG	ANG_09	0.169238	second-degree
RUS	RUS_11	RUS	RUS_04	0.120962	second-degree
ANG	ANG_10	ANG	ANG_07	0.107917	second-degree
ANG	ANG_09	ANG	ANG_07	0.103653	second-degree
BR	BR_02	BR	BR_01	0.103425	second-degree
ANG	ANG_10	ANG	ANG_06	0.102982	second-degree
BR	BR_04	BR	BR_01	0.101133	second-degree

**Table A5:** Results of the Tukey pairwise comparison to detect differences in observed heterozygosity (H_O_), expected heterozygosity (H_E_) and the inbreeding coefficient (*F*) between the three genetic groups *O. pannosa* subsp. *pannosa, Olearia* (FR) and *O. pannosa* subsp. *Cardiophylla.* Diff is the difference between means of the two groups, lwr and upr are the ends of the 95% confidence interval and p adj is the *p*-value after adjusting for the comparisons.

	Group Comparison	diff	lwr	upr	*p* adj
H_O_	*O*. *pannosa* subsp. *cardiophylla—Olearia* (FR)	−0.02	−0.07	0.04	0.68
	*O*. *pannosa* subsp. *pannosa—Olearia* (FR)	0.05	0	0.1	0.05
	*O*. *pannosa* subsp. *pannosa—O*. *pannosa* subsp. *cardiophylla*	0.07	0.04	0.1	<0.001
H_E_	O. *pannosa* subsp. *cardiophylla—Olearia* (FR)	−0.05	−0.14	0.04	0.32
	*O*. *pannosa* subsp. *pannosa—Olearia* (FR)	0.06	−0.03	0.14	0.25
	*O*. *pannosa* subsp. *pannosa—O*. *pannosa* subsp. *cardiophylla*	0.11	0.06	0.16	<0.001
*F*	*O*. *pannosa* subsp. *cardiophylla—Olearia* (FR)	−0.13	−0.23	−0.02	0.02
	*O. pannosa* subsp. *pannosa—Olearia* (FR)	−0.04	−0.14	0.07	0.64
	O. *pannosa* subsp. *pannosa—O*. *pannosa* subsp. *cardiophylla*	0.09	0.03	0.15	<0.001
Kinship	*O*. *pannosa* subsp. *cardiophylla—Olearia* (FR)	0.10	−0.10	0.30	0.42
	O. *pannosa* subsp. *pannosa—Olearia* (FR)	−0.02	−0.22	0.17	0.95
	*O*. *pannosa* subsp. *pannosa—O*. *pannosa* subsp. *cardiophylla*	−0.12	−0.23	−0.02	0.02

**Table A6:** Results of the one-way analysis of variance (ANOVA) conducted to detect differences in leaf shape between the three genetic groups *O. pannosa* subsp. *pannosa, Olearia* (FR), and *O. pannosa* subsp. *cardiophylla.* We have compared the differences between PC1 (width, length, and leaf surface area), and PC2 (ovality and location of widest point of the leaf).

	Group Comparison	diff	lwr	upr	*p* adj
PC1	*O. pannosa* subsp. *cardiophylla—Olearia* (FR)	0.68	−0.18	1.54	0.15
	*O. pannosa* subsp. *pannosa—Olearia* (FR)	2.45	1.74	1.74	<0.001
	*O. pannosa* subsp. *pannosa—O. pannosa* subsp. *cardiophylla*	3.13	2.42	3.84	<0.001
PC2	*O. pannosa* subsp. *cardiophylla—Olearia* (FR)	1.94	1.33	2.56	<0.001
	*O. pannosa* subsp. *pannosa—Olearia* (FR)	0.11	−0.39	0.62	0.86
	*O. pannosa* subsp. *pannosa—O. pannosa* subsp. *cardiophylla*	−1.83	−2.33	−1.32	<0.001
